# Synchronisation of parental behaviours reduces the risk of nest predation in a socially monogamous passerine bird

**DOI:** 10.1038/s41598-018-25746-5

**Published:** 2018-05-09

**Authors:** K. Leniowski, E. Węgrzyn

**Affiliations:** 1 Laboratory of Bioacoustics and Spectrophotometry, Faculty of Biotechnology, University of Rzeszów, Rejtana 16c, 35-959 Rzeszów, Poland; 2 Department of Zoology, Faculty of Biotechnology, University of Rzeszów, Rejtana 16c, 35-959 Rzeszów, Poland

## Abstract

Social monogamy with bi-parental care is the most common breeding pattern in birds, yet cooperation between mates has not been intensively studied to date. In this study we investigate synchronisation of parental behaviours in the blackcap *Sylvia atricapilla*, a species characterized by bi-parental care and high nest predation. We test the hypothesis that mates synchronize their behaviours to decrease total activity at the nest, which is known to affect predation rate in birds. We examine if blackcap parents synchronise their feeding trips more when nestlings are at the poikilothermic stage, and they may be more vulnerable to nest predation due to their inability to escape and survive outside the nest without parental brooding. We also investigate the alternation of feeding trips by parents. We show that blackcap parents synchronise the majority of their feeding trips during the whole nestling period, and the level of parental synchrony is higher before nestlings develop endothermy. The alternation of male and female feeding trips was much higher than would be expected by chance and was positively related to parental synchrony. We have demonstrated that synchronisation of parental feeding trips significantly decreased parental activity at the nest, and nest survival time increased with the synchrony of parental feeding trips.

## Introduction

Social monogamy occurs in 92% of bird species^1^ but it is not necessarily associated with both parents involved in the raising of offspring. Bi-parental care occurs in 75% of bird species^2^. Although parents of such species cooperate to raise young, the degree to which specific activities are coordinated to achieve this has not been well studied. Only recently have a few papers revealed that behavioural compatibility and synchronization constitute important aspects of parental care^3,4^ and may affect breeding success independently of the genetic quality of parents^5,6^. For example, pairs with better incubation coordination, measured by periods during which only one parent at a time attended the eggs, hatched more eggs^5^, while synchronization of feeding trips is positively related to brood size at hatching and the number of offspring in the nest a few days prior to fledging^3,7^. Because the benefits of each mate’s investment may be higher if partners synchronize their efforts with each other, such behaviours should be favoured by natural selection and widespread in those species with bi-parental care.

Nest predation is a major source of reproductive failure in birds^8,9^, and parental feeding trips are risky because they are likely to reveal the location of the nest to potential predators^10^. One of the most apparent ways to reduce activity around the nest is to synchronize parental behaviours^11^, as the simultaneity of mates’ movements may cut down the activity level at the nest by up to 50% depending on the level of matching. Additionally, synchronization of parental behaviours may reduce sexual conflict and its potential fitness costs for nestlings, because it allows each parent to control the investment and also to accurately monitor the effort of its mate^12^. For example, an individual may withhold provisioning until the partner has provisioned, which leads to alternation of provisioning^13^ and more equal distribution of work between parents. This kind of conditional cooperation requires integration of information about the mate’s investment, which may be obtained indirectly from nestling condition^14^ or directly from watching their partner. In the latter case, a high level of synchronization may be expected because it facilitates monitoring of their partner. The two adaptive explanations (i.e. reduction of nest predation and reduction of sexual conflict) are not mutually exclusive, and may have a combined effect in shaping parental coordination.

The subject of this study was the blackcap *Sylvia atricapilla*, a species characterized by bi-parental care. Nestlings are typically fed and brooded by both parents, and males develop brood patches similar to that of females (pers. observ.)^15,16^. The species is also subjected to high nest predation – on average only 30% of nests survive^17,18^. Thus, blackcaps constitute a good model for studying parental behavioural coordination. Synchronization between individuals may consist of (i) exhibiting the same behaviour at the same time (activity synchrony) or (ii) switching actions at the same time (temporal synchrony)^19^. Both kinds of synchronization may take place during brood rearing in altricial birds. Pertinently, an earlier study^20^ revealed that blackcap nestlings develop endothermy at the age of 7 days, and before this stage they require substantial brooding. Coordinated brooding and feeding in young poikilothermic hatchlings would thus involve switch-over behaviours at the nest. Simultaneous feeding trips performed by parents of endothermic nestlings would in turn represent the same behaviour at the same time. Here, we investigate both aspects of synchronization. We test the hypothesis that blackcap parents synchronize their behaviours to decrease disturbance at the nest caused by parental activity, which is known to affect nest predation rate in numerous bird species^10^. We examine if blackcap parents put more effort into synchronising their feeding trips to reduce disturbance at the nest at the stage of poikilothermic nestlings, which may be more vulnerable to predation. Higher vulnerability may result from the fact that poikilothermic nestlings cannot escape from the nest when attacked by a predator because they need parental brooding and therefore they are unable to survive outside the nest. We also examine whether synchronisation of parental activity affects nest survival. Finally, we investigate the alternation of feeding trips by parents and subsequently test the relationship between alternation and synchrony. Blackcaps nest in shrubs^17^ and forage in rather dense vegetation up to several dozen meters from the nest (pers. observ.) thus the visibility of a partner is poor if mates forage separately. As non-random alternation of provisioning requires the monitoring of a mate we assume that alternation of feeding trips will increase with parental synchrony.

## Results

### Parental synchronisation during the poikilothermic stage of nestlings

**Figure 1:**
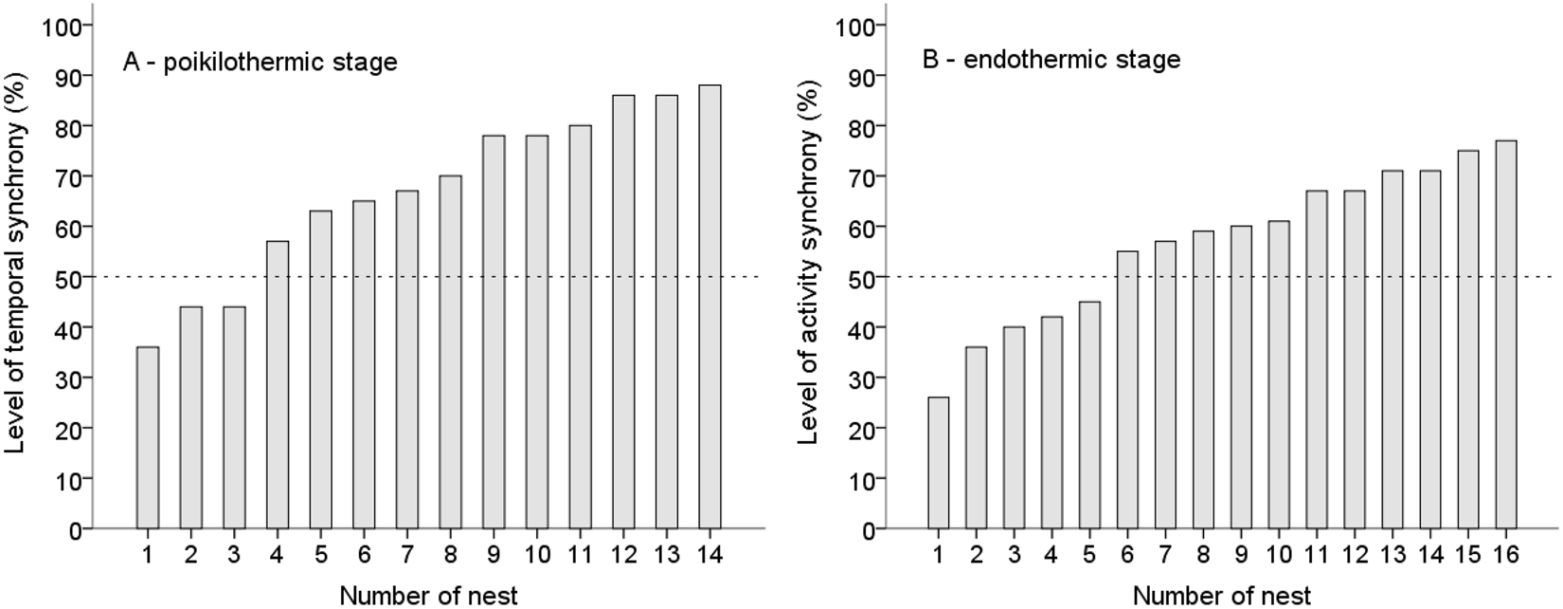
Behavioural synchronisation of Blackcap parents. (**A**) Temporal synchronisation during the poikilothermic nestling stage, n = 14 nests; (**B**) Activity synchronisation during the endothermic nestling stage, n = 16 nests. The level of synchronicity was presented for each studied nest. Nests were ranked from lowest to highest level of synchronicity. The dotted line indicates that two-thirds of pairs synchronised over 50% of feeding trips.

We found a high level of synchronisation of parental behaviours during the poikilothermic stage of nestlings (i.e. when mates took turns in feeding and brooding hatchlings and the lag between one parent’s departure from the nest and the other parent’s arrival to the nest was no longer than 60 sec). Parents performed more coordinated switches per hour (6.6 ± 2.8) than unsynchronised feeding trips (2.4 ± 1.3) and the difference was highly significant (t = −5.23, df = 13, p < 0.001). The arrival of one parent was synchronised with the departure of its mate on average in 67% ± 16.7 of all cases. A high level of synchronisation was characteristic for the majority of blackcap pairs - two-thirds of pairs synchronised over 50% of feeding trips (Fig. 1A). Comparison of the real disturbance at the nest (RD – caused by both synchronous and asynchronous parental feeding trips) to the theoretical maximum disturbance (TMD - that would be generated if all parental feeding trips were asynchronous), revealed that the reduction of the disturbance at the nest (RoD) caused by parental synchrony was 36% ± 7.8. Asynchronous feeding trips did not significantly affect the overall disturbance at the nest (LMM: F = 3.303, df = 4, p = 0.082). This may be explained by the fact that the number of asynchronous feeding trips was low – on average parents performed less than 3 such feeding trips per hour.

### Parental synchronisation during the endothermic stage of nestlings

Development of nestling endothermy caused changes in parental behaviour, as nestlings no longer depended on being brooded. Endothermic nestlings required more intensive feeding (mean feeding rate = 24.9 ± 8.07) than poikilothermic ones (7.3/h ± 3.5) and parental feeding trips significantly increased (t = 6.9, df = 28, p < 0.001). Although two-thirds of pairs still synchronised over 50% of feeding trips (Fig. 1B), the level of synchronisation was slightly lower (57% ± 15.07) than during the poikilothermic stage (67% ± 16.7). The number of asynchronous feeding trips increased and the difference between synchronous (13.9 ± 5.5) and asynchronous feeding trips (11.1 ± 6.0) was non-significant (t = −1.37, df = 15, p = 0.192). The reduction of disturbance at the nest (RoD) due to synchronous feeding trips was 28% ± 7.5. However, asynchronous feeding trips were more frequent than during the poikilothermic nestling stage (11.1 vs. 2.4 flights/h) and their effect on the disturbance at the nest became significant (LMM: F = 24.28, df = 11, p < 0.001).

### Parental synchrony, disturbance at the nest and nest survival

**Figure 2:**
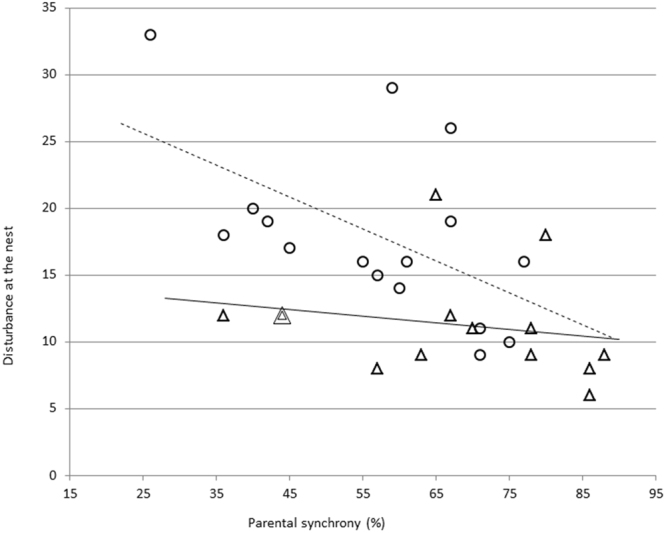
Relationship between the synchrony of parental feeding flights and the disturbance at the nest during the poikilothermic nestling stage (∆, n = 14 nests) and the endothermic nestling stage (○, n = 16 nests). Overlapping points are indicated by concentric circles and/or triangles (the number of symbols represents the number of overlapping points). The fitted lines serve for visualization purposes, not for statistical inference.

**Figure 3:**
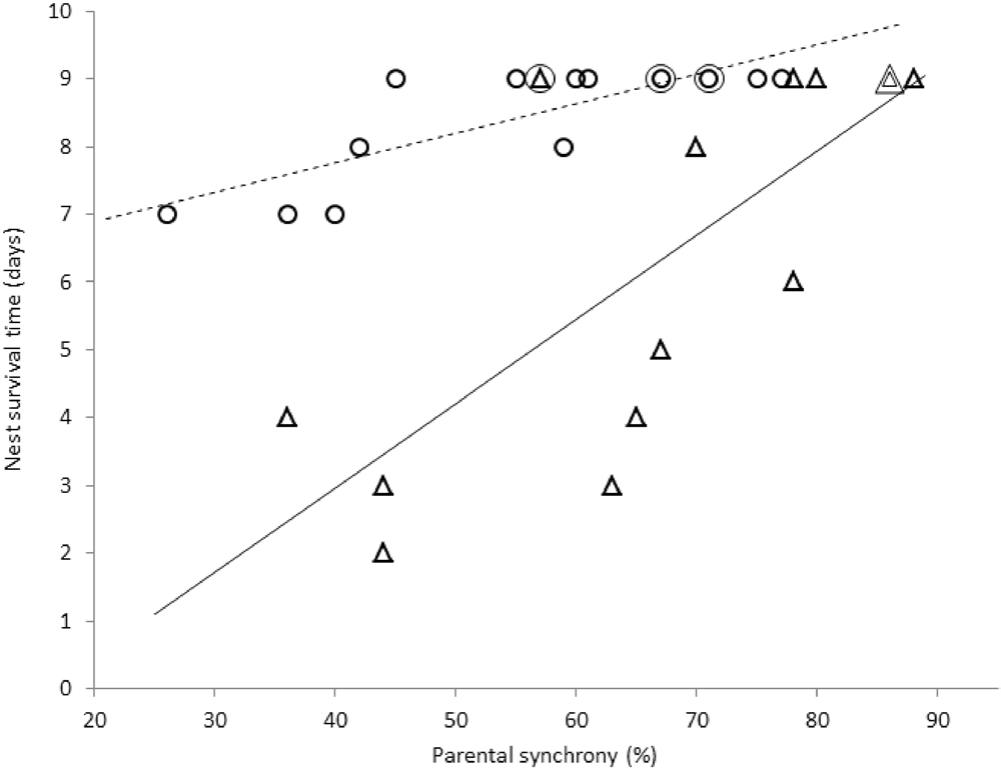
Relationship between the synchrony of parental feeding flights and the nest survival time during the poikilothermic nestling stage (∆, n = 14 nests) and the endothermic nestling stage (○, n = 16 nests). Overlapping points are indicated by concentric circles and/or triangles (the number of symbols represents the number of overlapping points). The fit lines serve for visualization purposes, not for statistical inference.

**Figure 4:**
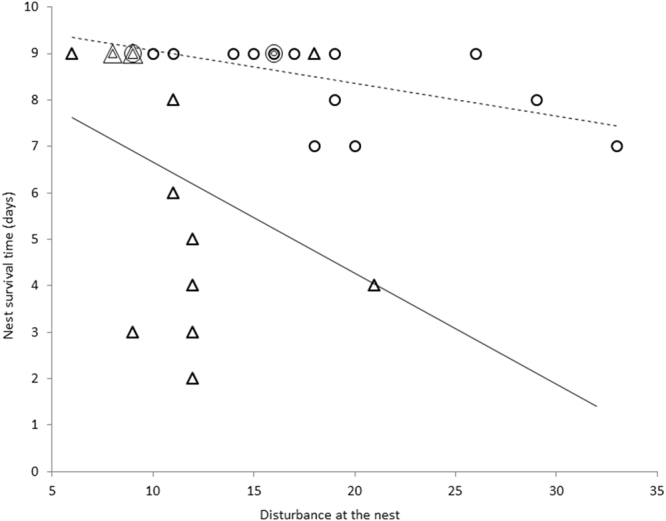
Relationship between the disturbance at the nest and the nest survival time during the poikilothermic nestling stage (∆, n = 14 nests) and the endothermic nestling stage (○, n = 16 nests). Overlapping points are indicated by concentric circles and/or triangles (the number of symbols represents the number of overlapping points). The fit lines serve for visualization purposes, not for statistical inference.

More synchronised parents caused less disturbance at the nest (Fig. 2). We found a significant negative correlation between parental synchrony and disturbance for the poikilothermic nestling stage (r_S_ = −0.52, p = 0.030, n = 14), the endothermic nestling stage (r_S_ = −0.59, p = 0.017, n = 16) and the whole nestling stage (r_S_ = −0.58, p < 0.001, n = 30). Nestlings provisioned by more synchronised parents tended to survive longer than nestlings of less synchronised mates (Fig. 3). There was a significant positive correlation between parental synchrony and nest survival time during the poikilothermic nestling stage (r_S_ = 0.77, p = 0.001, n = 14), the endothermic nestling stage (r_S_ = 0.75, p = 0.001, n = 16) and the whole nestling stage (r_S_ = 0.5, p = 0.003, n = 30). At the same time there was no significant relationship between parental feeding rate and nest survival time during the poikilothermic nestling stage (r_S_ = −0.34, p = 0.12, n = 14) and a near-significant relationship during the endothermic nestling stage (r_S_ = −0.42, p = 0.052, n = 16). There was, however, a significant negative correlation between the disturbance around the nest caused by parental provisioning and nest survival time (Fig. 4) both during the poikilothermic nestling stage (r_S_ = −0.56, p = 0.020, n = 14) and during the endothermic nestling stage (r_S_ = −0.66, p = 0.003, n = 16).

### Alternation of feeding trips

**Figure 5:**
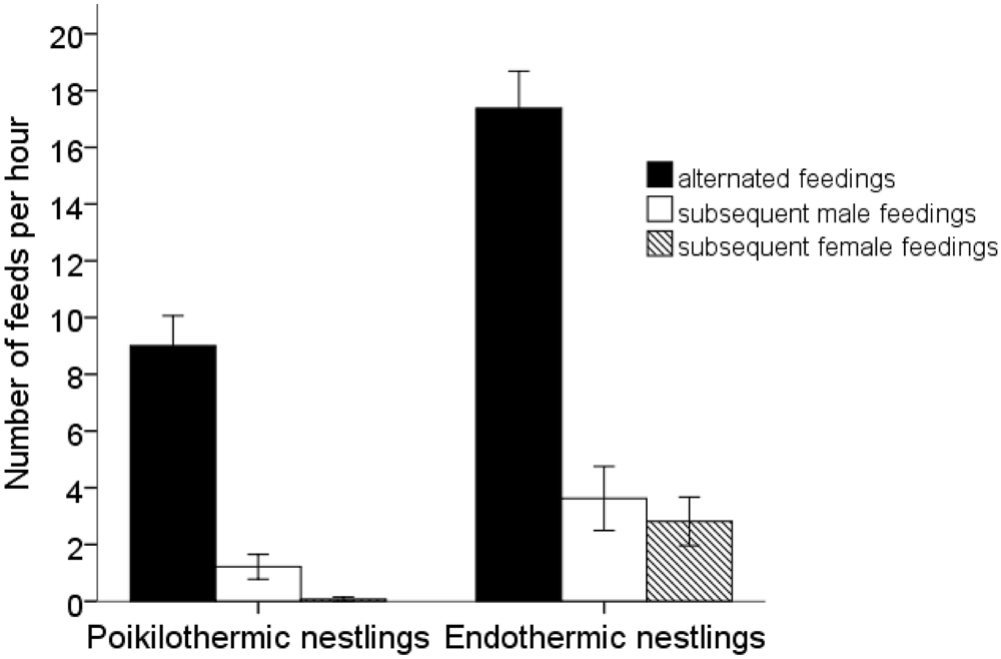
The ratio of alternated parental feeding trips and feeding trips performed subsequently by males and females in nests containing poikilothermic nestlings (n = 14) and endothermic nestlings (n = 16), whiskers represent 1 SE.

The alternation performed by parents of poikilothermic (88.0% ± 10.84) and endothermic (75.3% ± 13.96) nestlings was much higher than would be expected by chance (24.5% ± 2.48 and 23.0% ± 2.95, respectively) and the difference between observed and expected alternation was highly significant both in poikilothermic (t = −24.1, df = 13, p < 0.001) and endothermic (t = 17.28, df = 15, p < 0.001) stages of nestling growth. Parents performed significantly more alternated than subsequent feeding trips during poikilothermic (t = 13.496, df = 13, p < 0.001) and endothermic nest stage (t = 7.243, df = 15, p < 0.001). The ratio of alternated parental feeding trips and feeding trips performed subsequently by males and females is shown in Fig. 5.

Alternation of provisioning significantly increased with parental synchronization (LMM: F = 6.63, df = 11, p = 0.003), but was not affected by nestling thermoregulation (LMM: F = 0.07, df = 1, p = 0.800) or provisioning rate differences between parents (LMM: F = 1.48, df = 7, p = 0.280).

## Discussion

The results of our study show that bird parents highly synchronise their behaviours during the whole nestling period, which highlights the importance of cooperation between mates. In the blackcap, which suffers from heavy nest predation (on average only 30% of nests survive)^17,18^, the synchronisation of parental feeding trips may have evolved as an anti-predator strategy because nest predation increases with disturbance around the nest caused by parental activity^10^. Synchronized behaviours can be very important for pooling nest visits to reduce the probability of the nest being detected by predators^11^. A previous study by Węgrzyn^20^ revealed that resource allocation between growth and endothermy enables rapid nestling development, which shortens nest exposure time to predators^21^. Reduction of parental activity at the nest due to synchronisation of feeding trips may further decrease the risk of nest predation. This is supported by this study which shows that nest survival time is positively related with parental synchrony.

Coordination of parental care may also improve nestling development through more effective brooding. A study of cockatiels *Nymphicus* hollandicus^5^ demonstrated that greater coordination of incubation improves egg hatchability. Synchronisation of brooding, when parents swap places with each other in a coordinated way, may have a positive effect on the development of poikilothermic nestlings, because it protects nestlings from cooling and reaching critically low temperature levels that may negatively affect their condition and survival^22.^ As the rate of development affects nestling exposure time to predators, synchronisation of brooding may thus constitute another aspect of an effective anti-predator strategy.

Synchronisation of parental behaviours may also decrease predation on adult birds. The parent which is currently in the nest has reduced visibility and its partner may act as a sentinel while waiting outside, as shown in the zebra finch^4^. Although blackcaps are open nesters they experience reduced visibility while in the nest due to foliage cover which provides nest concealment. A well-concealed nest is safer because of reduced transmission of auditory, visual and olfactory cues from the nest to potential predators^9^ but simultaneously the parent in the nest suffers from reduced visibility of nest surroundings. A sentinel partner may thus warn about approaching predators. Nest visit synchronization is therefore likely to reduce predation of both offspring and adults around the nest by acting as sentinels. As a result we may expect strong selection on coordinated patterns of parental care.

Despite the fact that synchronisation of parental care in birds seems to have multiple beneficial effects and may be widespread among bi-parental species, studies on this subject are rather scarce. Only recently has it been found that in bi-parental species the reproductive success of a pair is at least partly determined by behavioural compatibility between partners^5,23–25^. A few other studies have investigated parental synchrony and reported findings similar to ours: in the crimson rosella *Platycercus elegans* synchronisation of parental visits in the nest was 63%^26^, in the zebra finch 78%^3^ and in the long-tailed finch, *Poephila acuticauda* 73%^7^. In our study blackcaps demonstrated synchronization from 57% to 67% depending on the nestling stage. The slightly lower level of parental synchronisation in blackcaps compared to the cited species may result from a much higher feeding rate in our study species: blackcaps performed between ten and twenty five feeding trips per hour while the other species about one feeding trip during the same time. Synchronisation may be easier at lower feeding rates because quickening the pace may disrupt the coordination of feeding trips with a partner. For example, if each of the two mates has got only two or three minutes to find a caterpillar and to perform a synchronised feeding trip with their partner, it may be more demanding than if each of them has got one hour for the same task. This may also explain why blackcap parents in our study were less synchronised while rearing endothermic nestlings. Endothermy involves doubling the feeding rate^20^, and it may be impossible for parents to reconcile such a food demand with high synchrony of nest visits. Alternatively the lower synchronisation observed in blackcaps rearing endothermic offspring may result from the fact that endothermic nestlings may be less vulnerable to predation because they can escape from the nest when a nest predator arrives. So parents may put less effort into minimising the risk of predation caused by their activity around the nest. The above premises are not mutually exclusive – it may be both more difficult to maintain synchronisation at higher feeding rates and less indispensable to do so when predation imposes a lower threat. It should be noted that due to rather limited sample sizes and the lack of some data we did not control for all possible factors that theoretically may have interacted with parental coordination, like for example nest concealment, the age of parents or predator identity. Thus, we cannot rule out the possibility that parental coordination observed in our study may also play another function or may have different reasons.

Apart from the potential of reducing the risk of predation, synchronisation of parental feeding trips may also have evolved in order to enable each parent to monitor its mate’s investment. Recent theoretical and empirical studies propose that partners may resolve sexual conflict over the level of parental care provided through negotiations over the division of labour^12,13^. One of the mechanisms used by parents to reduce conflict may be the alternation of feeding trips, which ensures that none of them free-rides on the other’s efforts. Interestingly, our study found that blackcap parents alternate their feeding trips significantly more often than would be expected by chance, and the alternation of provisioning significantly increased with parental synchronization. As high level of alternation is hardly possible without knowledge of the partner’s actions, synchronised flights during provisioning may be the simplest way to monitor a mate and to make sure that the investment of each parent is equal. Thus it seems likely that synchronisation of feeding trips in connection with alternation of provisioning may constitute a mechanism that reduces sexual conflict. Similar findings were presented by Bebbington & Hatchwell^12^ in long-tailed tits *Aegithalos caudatus*, although synchronisation occurred at a much lower rate in their study (13% compared to 57–67% in ours). Also, alternation was affected by the provisioning rate difference between long-tailed tit parents, while in our study we did not find such an effect. Both differences between studies may be explained by the fact that blackcap parents mostly provisioned at similar rates, which enabled better synchronisation and did not affect alternation. Interestingly, Bebbington & Hatchwell^12^ did not find any fitness correlates of parental coordination except for the fact that alternation in successful nests was significantly higher than in depredated nests. As alternation was positively related to synchrony of feeding trips, it was suggested that coordinated flights reduced the disturbance around the nest, which is known to attract predators^11,27^. This is in accordance with the conclusions of our study of blackcaps.

Our study highlights the importance of synchronisation of parental behaviours in bi-parental species. We show that parents synchronise the majority of their actions during the whole nestling period, despite the fact that the form of their cooperation is different when nestlings are poikilothermic and endothermic. Mates switch from temporal synchrony to activity synchrony to adjust their care to current nestling demands and maintain a high level of coordination at the same time. This clear tendency for continuous synchronization of parental care over the whole nestling period suggests the importance of such behaviour. It has the potential to reduce the nest predation rate as well as sexual conflict between parents, through facilitation of alternated feeding trips. More studies are needed to explore other benefits gained from parental coordination.

## Methods

### Species and study area

Data were collected in 2009–2014 in the deciduous forest of the Fox Hill Reserve on the outskirts of Rzeszow, south-eastern Poland (DD: 50.009567, 21.987223).

The blackcap is a small, migratory, open-nesting passerine that breeds in central Europe. It prefers habitats characterized by dense tree and shrub vegetation^28^. It builds thin-walled, open cup nests in the shrub and herbaceous layers of forests^17^. The clutch size is three to six eggs laid on consecutive days. Incubation lasts about 12 days and nestlings stay in the nest for another 12 days, but they are able to leave the nest when 9 days old. Nestlings develop endothermy at the age of 7 days^20^ and brooding decreases significantly at this age^29^. The blackcap suffers from high rates of nest predation, mostly by corvids^30,31^. On average only 30% of nests escape predation (nest success reported from different countries: 20% - Germany^30^, 31% - Czech Republic^18^, 42–61% - Slovakia^32^, 20–49% - Poland^17,33^). Similar nest success (34%) was observed in our study site^20^. In the blackcap the main predators seem to be corvids (jays in particular)^30^ and the majority of loses are due to daytime predation events. In two of eight years of our project we checked nests just after dawn and before dusk and we recorded only three nest loses during the night. Two of them resulted from a heavy night storm (both nests were lost during the same night and nestlings were found dead under the nest) and the third one was predated as nestlings were missing and the nest was torn. This shows that night nest predation is not common in blackcaps, at least in our study area. Also, the majority of nests predated during the day time are left intact, suggesting aerial predator as a major cause of loses.

### Data collection

Nests were found by careful inspection of potential nest-sites after mapping male breeding territories in the spring. We searched for nests from mid-April until the end of June in every year of the study using the nest monitoring protocol described in Węgrzyn^20^. Despite the fact that we used recordings collected during 6 breeding seasons there is a very little chance that the same pair was sampled more than once. First, life expectancy of blackcaps is only 2 years^34^, the adult annual survival rate is 43% (males 46%, females 29%). As songbirds typically do not extend a pair-bond over a breeding season it seems unlikely that we recorded the same pair in subsequent seasons, especially taking into consideration low survival rates of blackcaps. It is important to note that we analysed the behaviour of a pair, not an individual. Even if the same male returned during the following season and he was filmed, it is not likely that he was paired with the same female. The same bird paired with a different partner creates a new pair and these two pairs are independent samples in the analyses. Additionally, blackcap males are characterised by high site-fidelity rate and all but five of our recordings came from different territories. Of the five recordings that were taken on the same territories, two were separated by 4 years and remaining three by 5 years, thus the probability that the same pairs were recorded is minimal. Taking all above facts into consideration we are positive that the probability that we recorded the same pairs in multiple years and our recordings are non-independent of each other in the analyses is very low.

### Synchronisation of parental behaviours

We quantified synchronisation of parental behaviours in 14 nests containing poikilothermic nestlings (aged 2–6 days), and 16 nests containing endothermic nestlings (aged 7–10 days). These observations represent 30 different nests and no nest contributed data to both stages. We classified nestlings according to their thermoregulatory ability because feeding rate is quite constant during the poikilothermic stage (nestlings 2–6 days old), after which it rapidly increases on day 7 (the onset of endothermy) and remains at this level for the rest of the endothermic stage^20^. Each nest was filmed for 1 hour (detailed description is presented further down in the Methods section entitled *Filming procedure*). Male and female identity was recognized based on sexual dimorphism between parents: the male has a black cap whereas the female has a brown cap.

We used a different approach to analyse parental synchronization depending on the thermal state of offspring. In nests with polikilothermic nestlings we investigated switching actions at the same time because blackcap parents swap places with each other in feeding and brooding at this stage of the nesting period^29^. Thus we considered parental behaviours as synchronized if one parent’s departure from the nest coincided with the other parent’s arrival and the lag between the two activities was no longer than 60 seconds. On many occasions parents swapped places at the nest directly but we also observed numerous cases when the arriving parent landed in close proximity to the nest and entered it soon after its mate had left. For this reason we treated all switches that happened within 1 minute as synchronised. During the poikilothermic stage each parent visits the nest about 4 times per hour. Given the duration of intervals between visits we believe that 1 minute is a justified lag and implies that parents switched at the nest in a coordinated way.

In nests with endothermic nestlings parents cease brooding, and if any synchronization occurs it should take form of simultaneous feeding trips. Again, we considered a parental feeding trip as synchronous if the lag between parents entering the nest was no longer than 60 seconds. We have numerous observations that blackcap parents often arrive with food and perch in the nest vicinity together before one of them enters the nest. The other mate waits and enters the nest after the first one has exited. We also noticed that in many cases the first bird after leaving the nest remained close until its partner joined them after feeding nestlings, then the pair flew away together. Based on the above description of synchrony we counted the number and the percentage of synchronous and asynchronous parental feeding trips for each nest during one hour video recording.

### Nest disturbance

The video recordings were also scored for the number of trips to and from the nest by both parents feeding nestlings. Such trips constitute the kind of parental activity at the nest that may draw the attention of a predator. Importantly, parental activity from this perspective should be considered as a disturbance caused by flight to or from the nest. Thus one trip by a single parent creates the same level of disturbance as one trip of synchronised mates. Consequently, at any given feeding rate the unsynchronised mates generate twice as high a disturbance as synchronised mates. To calculate the disturbance around the nest during 1 hour of video recording, each unsynchronised parental trip was scored 1 and each synchronised trip by mates was also scored 1 (so if parents flew synchronously each of them received ½ a point for a trip). Thus one parent for one unsynchronised feeding trip (arrival and departure) scored 2 points, and one parent for one synchronised feeding trip (arrival and departure) scored 1 point. As a result, the more synchronised feeding trips the lower total disturbance at the nest.

To calculate the reduction of disturbance due to parental synchrony we first calculated a theoretical maximum disturbance (TMD) at the nest which would occur if all observed visits at the nest were unsynchronised (each parental feeding trip was scored 2 points: 1 point for arrival to the nest and 1 point for departure from the nest). Next, we calculated a real disturbance (RD) as the sum of points for both unsynchronised (2 points) and synchronised (1 point) feeding trips by each parent. Finally we calculated the reduction of the disturbance (RoD) using equation: RD/TMD × 100%. For example, if both mates (male and female) performed 10 feeding trips/h each and all feeding trips were synchronous (RD = 10 but TMD = 20), they would reduce disturbance at the nest by 50% (calculated as: 10/20 × 100%).

### Alternation of feeding trips

Alternation, A, was calculated as A = F/(t − 1), where *F* is the number of times a parent fed after its mate and *t* is the total number of feeds during 1 h observation^10^. As some alternation will occur by chance even if parents do not perform it intentionally, we calculated the expected alternation for each nest and we compared it to the observed alternation using a paired sample t-test. To calculate the expected alternation of each pair, we first estimated male and female share in all parental feeding trips during 1 hour of video recording. This was needed as the probability of alternation which occurs by chance depends on the male and female share in feeding. Then, using the theory of probability, for each pair we simulated 10000 approaches of parents to the nest, maintaining the ratio of original male and female feeding trips in each pair. Next, we checked how many of simulated 10000 feeding trips were alternated and we calculated the proportion of alternate feeding trips to all feeding trips. 10000 feeding approaches were simulated using sampling without replacement. For each pair we prepared an initial pool of 10000 parental feeding trips containing male and female feeding trips in the original proportion of a given pair. The simulation was based on bimodal randomisation method with respect to male and female share in feeding trips (program code is given in the Appendix). As result, we received bimodal probability distribution of feeding trips with the sum of 10000 approaches. Finally, we estimated the level of alternated feeding trips (i. e. when the subsequently sampled feeding trips belonged to different sexes). For each pair we also calculated provisioning rate difference as the difference between female and male feeding rates, as this variable also has the potential to influence alternation of provisioning (parents feeding at different feeding rates cannot achieve 100% alternation).

### Nest survival

To investigate the effect of parental synchronisation on nest survival we used the nest survival time (NST) as the number of days that a nest survived since nestling hatching to predation or the ability to fledge. A nest was considered successful if it survived 9 days (because nestlings are ready to fledge at any time since this age if disturbed and the brood as a whole is not vulnerable to predation). Thus we used 9 days as the upper nest age limit in analyses of nest survival, irrespective of the real time of fledging (up to 13 days since hatching). As a consequence, the survival time of all predated nests was the number of days since hatching (day 1) to predation and the survival time of all successful nests was 9 days.

### Relation between feeding rate and parental coordination

Although both synchronisation and alternation theoretically may increase with feeding rate (because at higher feeding rates there may be a higher chance that both parents feed within 60 seconds), we think that it is not the case in our study. Feeding rate potentially affects synchronisation and alternation of feeding trips only when time lags between subsequent parental feeding trips (of both parents) are constant and each parent performs the same number of feeding trips per hour. But in our study the time lags between subsequent feeding trips of a particular parent varied greatly and the number of male and female feeding trips per hour was not equal. To illustrate the real course of parental feeding trips we present the analysis of 1 hour of provisioning in a randomly chosen endothermic nest from our study (we used a nest of endothermic nestlings because during this stage feeding rate was the highest): altogether 39 feeding tripss, 21 by the male and 17 by the female, mean time lag between male feeding trips: 153 seconds (min = 24 seconds, max = 390 seconds, SD = 92.7), mean time lag between female feeding trips: 199 seconds (min = 25 seconds, max = 385 seconds, SD = 108.9). Obviously one parent was able to return to the nest multiple times while the other was searching for food because minimum time lag between feeding trips was about 24–25 s for both sexes and maximum about 385–390 s. Thus we assume that to keep synchronisation and/or alternation of feeding trips, parents had to actively adjust their feeding trips to each other. Additionally, there was no significant correlation between parental synchronisation and feeding rate during the poikilothermic nestling stage (r_S_ = −0.34, p = 0.12, n = 14) and the endothermic nestling stage (r_S_ = −0.27, p = 0.16, n = 16), demonstrating that parents who provision more often are not more synchronous.

### Filming procedure

A micro-camera (of thumb-nail size) was placed about 25 cm from the nest and left for 1 h to allow parents to resume their natural feeding activity. We used the micro-camera (Sony 1/4′′ CCD matrix, pinhole lens; Tokyo, Japan) connected to a laptop through a Pinnacle Studio 10 USB video converter. For a more detailed description of camera mounting refer to Węgrzyn & Leniowski^29^. Before we started our project on blackcaps we had tested how long it takes blackcap parents to resume their natural feeding rate after mounting a camera. To do this we estimated feeding rate in 10 nests before mounting a camera by watching the nests from a distance, using binoculars or a telescope. Next we mounted a camera and checked times after which parents resumed feeding rates observed before a camera was mounted. Tested pairs resumed feeding rate within 10–40 minutes, depending on a pair. Thus we decided that 1 hour is a sufficient time for blackcaps to resume natural behaviours at the nest, and we used such approach in all subsequent studies, including the current one. After parents resumed their natural feeding trips, each nest was filmed for 1 h. All recordings were made between 6.00 a.m. and 9.00 a.m. Time of the recording did not affect parental feeding rate, both in nests with poikilothermic nestlings (ANOVA F = 2.82, p = 0.1, df_1_ = 2, df_2_ = 11, n = 14 nests, effect size: η^2^ = 0.34) and endothermic nestlings (ANOVA F = 1.94, p = 0.18, df_1_ = 2, df_2_ = 13, n = 16 nests, effect size: η^2^ = 0.23).

### Statistical analyses

All analyses were conducted using SPSS version 20 software (SPSS Inc., Chicago) with an α value of 0.05 used to denote significance. When reporting value, the mean ± SD notation is used. The effect of asynchronous feeding trips on the disturbance at the nest was tested with a Linear Mixed Model (LMM) with disturbance at the nest as a dependent variable, the number of parental asynchronous feeding trips as a fixed factor and the year as a random factor (to control for this variable). To investigate potential predictors of alternation at a nest we used a LMM with the number of alternated provisioning visits as a dependant variable, the number of parental synchronous feeding trips, nestling thermoregulation stage and the provisioning rate difference between parents as fixed factors and year as a random factor (to control for this variable). Relation between parental synchrony and nest survival time were checked using Spearman correlation. The same analysis was used to test the relation between: feeding rate and parental synchrony, parental synchrony and disturbance at the nest, disturbance at the nest and nest survival time.

To test if the real disturbance at the nest (RD) was significantly lower than the theoretical maximum disturbance (TMD) we compared RD and TMD calculated for each nest using a paired T-test. The same test was used to compare the difference between observed and expected alternation as well as the difference between alternated feeding trips and subsequent feeding trips performed by the same parent. To compare the difference between parental feeding trips to poikilothermic and endothermic nestlings we used an independent samples T-test. The same test was used to analyse the difference between synchronised and unsynchronised feeding trips in nests containing poikilothermic and endothermic nestlings.

### Compliance With Ethical Standards

The study does not involve experiments on live vertebrates. All video recordings were made with minimum disturbance to the nests and they did not cause the desertion of any nests or behavioural changes in parental activity (nests were observed from the distance using binoculars or a telescope on the subsequent day after filming and we didn’t observe any abnormalities in parental behaviours).

## Electronic supplementary material

Appendix 1
