# Supplementary material for: Synchronisation of parental behaviours reduces the risk of nest predation in a socially monogamous passerine bird
Source: Sci Rep. 2018 May 9;8:7385. doi: 10.1038/s41598-018-25746-5 (PMC5943351; doi:10.1038/s41598-018-25746-5)
Supplement: Supplementary file 1 — Appendix 1 [file 41598_2018_25746_MOESM1_ESM.pdf]

Title page:

Synchronisation of parental behaviours reduces the risk of nest predation in a socially monogamous passerine bird

*K. Leniowski[1] & E. Węgrzyn[2]**

[1] Laboratory of Bioacoustics and Spectrophotometry, Faculty of Biotechnology, University of Rzeszów, Rejtana 16c, 35-959 Rzeszów, Poland

[2] Department of Zoology, Faculty of Biotechnology, University of Rzeszów, Rejtana 16c, 35-959 Rzeszów, Poland

*Corresponding author e-mail: songbird.ewa@gmail.com

Appendix

Program code (HaskellPlatform) used to calculate the expected alternation for each nest

(Mersenne.Pure64 library needed):

```
module Main where

import Data.List
import System.Environment
import System.Random
import System.Random.Mersenne.Pure64

line' [] acc = acc
line' [a] acc = acc
line' (x : y : ls) acc | x == y    = line' ls acc
                       | otherwise = line' ls $ acc + 1

line'' t a b = replicate na 0 ++ replicate nb 1 ++ last
  where
    (fa, fb, ft) = (fromIntegral a, fromIntegral b,
fromIntegral t)
    pa = ft * (fa/(fa+fb))
    pb = ft * (fb/(fa+fb))
    na = floor pa
    nb = floor pb
    last = if na + nb == t then []
           else map snd $ tail $ sort [(pa - fromIntegral na,
0),(pb - fromIntegral nb, 1)]

shu mt ls = map snd $ sort $ snd $ mapAccumR f mt ls
  where
    f mt x = (mt', (r, x)) where (r, mt') = randomDouble mt

line t (a : b : []) = do
  mt <- newPureMT
  putStrLn $ show $ flip line' 0 $ shu mt $ line'' t a b
line t _ = putStrLn "Could not parse the line!"

main = do
  args <- getArgs
  let trails = (sum $ map read args)
  if trails > 0 then
    getContents >>= mapM_ (line trails . map read . words) .
lines
  else
    putStrLn "Wrong number of trails, use a strictly positive
natural number!"
```
